# Management of intraoperative acetabular fracture in primary total hip arthroplasty

**DOI:** 10.1186/s12891-020-03356-5

**Published:** 2020-06-15

**Authors:** Juncheng Li, Quanbo Ji, Ming Ni, Qingyuan Zheng, Jingyang Sun, Guoqiang Zhang

**Affiliations:** Department of Orthopedic Surgery, Chinese People’s Liberation Army General Hospital, 28 Fuxing Road, Beijing, 100853 People’s Republic of China

**Keywords:** Primary total hip arthroplasty, Intraoperative acetabular fracture, Ankylosing spondylitis involving hip joints

## Abstract

**Background:**

Intraoperative acetabular fracture(IAF) is a rare complication of primary total hip arthroplasty(THA). The previous reports have lacked a sufficiently large number of subjects to allow for an analysis of the causes and appropriate treatment of this problem.

**Methods:**

Between 2015 to 2018, 4888 primary THA were enrolled. We retrospectively reviewed the records in our Total Joint Registry Database and found that 24 patients (24 hips) had sustained intraoperative acetabular fractures. Twenty-four patients(16 females and 8males)were all treated with a posterolateral approach using uncemented components. Twenty patients(83.3%)underwent supplemental screw fixation, of which 2 patients were treated with steel plate fixation. Two patients’ femoral heads were used as a graft. In 4 patients(16.7%), the acetabular components were judged to be stable despite the fracture and no additional treatment was performed. All patients were evaluated clinically with Harris Hip Scores (HHS) and radiographically with serial X-rays which follow up for a mean period of 34.0 ± 12.6 months. We evaluated the anatomic locations, causes, treatments, and outcome of the fractures to study the treatment method and effect of intraoperative acetabular fracture during operation.

**Results:**

The fracture rate associated with uncemented components was 0.49%. In 17(70.8%) of these patients, the fracture was noted during the impaction of the real acetabular component. Six patients(25%)with Ankylosing Spondylitis had fractures, 4 in the anterior wall, and 1 in the anterior column, because the patient with hip joint fusion needs a to pre-osteotomy before the dislocation. The HHS score increased from 30.8 ± 9.7 preoperatively to 90.2 ± 4.2 postoperatively. All the latest x-ray showed that the fracture did not move, and there is no translucent line formed in the acetabular cup bone interface.

**Conclusion:**

Intraoperative acetabular fractures are rare complications of THA, and most commonly occur during the implantation of the acetabular components. It is necessary to prevent the occurrence of fractures as much as possible even if the fractures are found during the operation. It should be noted that patients with ankylosing spondylitis involving hip joints during THA surgery must be careful to prevent IAFs during dislocation and pre-osteotomy.

## Background

IAF is a rare complication of the primary total hip arthroplasty (THA) [1–5]. Because of their concealment, they are more likely to be converted to postoperative complications [6]. As the number of uncemented acetabulum increases, the complications such as Intraoperative acetabular fracture (IAF) will continue to grow [7, 8]. Currently, there is a paucity of population-based studies on periprosthetic acetabular fractures, thus the true prevalence and incidence of these fractures remain unknown. Haidukewych and colleagues [6] found that in the past 10 years, the incidence of IAF with the use of uncemented components in 7121 patients who underwent the primary total hip arthroplasty was 0.4%. Such a fracture can occur during acetabular exposure, hip dislocation, reaming of the acetabulum, or impaction of the acetabular component. Previous studies have shown that under-reaming of the acetabulum and impaction of a relatively large acetabular component may predispose to intraoperative fracture [1, 9–11]. Good fracture healing and uneventful osseous ingrowth can be obtained with appropriate treatment such as weight-bearing restriction, change of the acetabular component, or addition of supplementary fixation screws, even if a fracture does occur [4, 6]. However, little has been written about the risk factors, treatment, and outcomes following this event. The purpose of the present study was to retrospectively review a larger consecutive series of patients who had sustained IAF of the acetabulum during primary THA.

## Methods

### Patients information

Between 2015 and 2018, 6 fellowship-trained arthroplasty surgeons performed 4888 uncemented primary THA in our institution. We reviewed the Total Joint Registry and found 24 cases (24 Hip) cases of acetabular fractures during the operation were judged by reviewing hospital database and postoperative X-rays. The average age of these patients (16 women, 8 men) was 53.8 ± 12.1 years (range, 35–78 years). The average of their weight is 64.5 ± 12.6 Kg (range, 40–85 Kg) and BMI is 25.2 ± 4.0Kg/m^2^(range, 16.9–34.3 Kg/m^2^). Of the 24 patients, the preoperative diagnoses include Adult avascular necrosis (11), ankylosing spondylitis (6), developmental dysplasia of the hip (5), osteoarthritis (2). The main symptoms of all patients were pain on the affected side and limited mobility. It affected life and performed the primary THA treatment.

### Follow-up evaluation

All patients were followed up by the surgeon after the operation for the X-ray examination and Hip Harris Score (HHS) after the operation. The information is obtained from the hospital database and confirmed by electronic medical records to obtain the cause and location of acetabular fractures. For acetabular fractures found after surgery, they can be obtained immediately after the operation of the X-rays. In the hospital database, we obtained whether to use screw fixation, plate fixation, bone grafting during the operation, and the period of fracture.

### Surgical technique

Twenty-four patients were all treated with the posterolateral approach. Twenty-four THAs adopt uncemented acetabular components and uncemented femoral components. The cohort’s acetabular components were 13 Pinnacle (Depuy Synthes),4 CombiCup SC (Link), 6 BetaCup (Link), 1 Trabeculae Orient Pattern (TOP Link). The cohort’s 24 femoral components consisted of 10 LCU (Link), 8 S-ROM combined prostheses (Deputu Synthes), 4 patients Corail (Deputu Synthes). 1 Ribbed (Link) and 1 BetaCone (Link).

In 20 cases, the acetabular component underwent supplemental screw fixation, of which 2 patients were treated with plate fixation. Two patients’ femoral heads were used as a graft. In 4 patients, the acetabular component was judged to be stable despite the fracture and no additional treatment was performed.

**Fig. 1:**
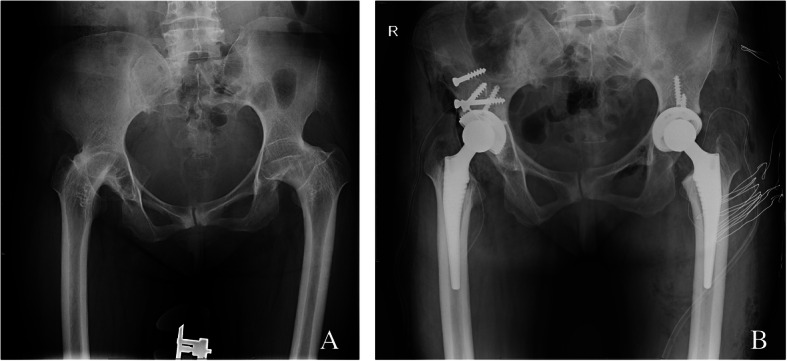
**a** Patient 1, Preoperative x-rays with double hip fusion. **b** The full-screw screws were nailed into the fracture ends, and the screw caps were replaced with steel wires in the form of cross-shaped bundling and compression

**Fig.2:**
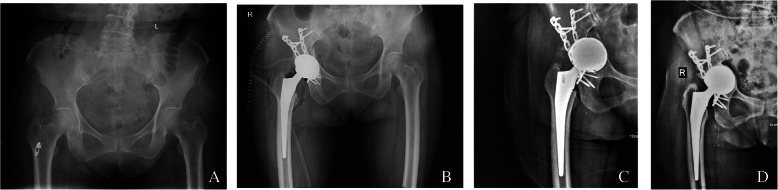
**a** Patient 5,Preoperative x-ray. **b** X-ray after operation for 3 days, posterior column fracture, internal fixation with steel plate screws. **c** X-ray 2 months after operation. **d** X-ray 3 years after operation, the component is stable

**Fig. 3:**
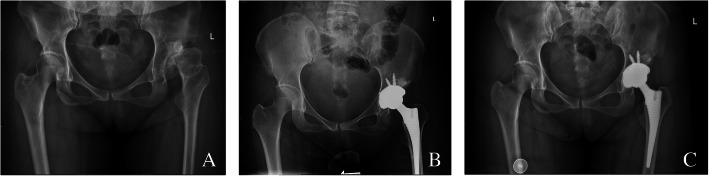
**a** Patient 17, severe DDH on the left side. **b** X-rays after operation for 3 days. **c** review after 3 year, the position of the component is stable

**Fig. 4:**
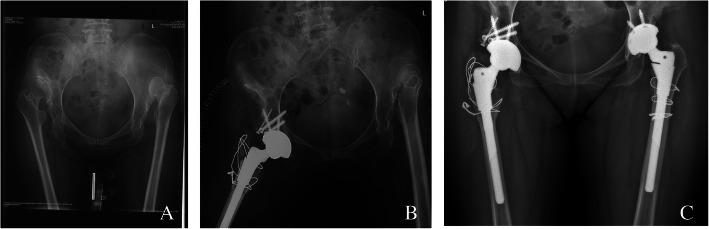
**a** Patient 20,Preoperative x-ray. **b** X-ray after operation 3 days.The femoral tuberosity was split during the operation and tied with steel wire. **c** X-ray after 2 year, the fracture heals

A patient with ankylosing spondylitis affected both hips, right femoral neck osteotomy was performed before hip dislocation, and anterior wall fractures occurred after the osteotome was inserted. After the Kirschner wire fixation and reduction, the three-point full-thread screw was fixed over the fracture line, but the reduction was not complete. The fractured end was loosened again, and the tension screw was fixed. The file was still loose, and then screwed with full-thread screws. At the fracture line, the nut is replaced with steel plates in the form of a steel wire cross bundling and compression, and the other parts are fixed with two screws to verify the stability (Fig. 1). A patient shows the acetabular medial wall, posterior wall, and posterior column when the acetabular component is installed during the operation. Fracture of the column was treated by internal fixation with plate and screws (Fig. 2). A patient shows the pendulum saw did not closely follow the tuberosity, and the osteotomy was elevated, which caused part of the anterior column to be sawed off. The pinnacle cup was stable. A patient with acetabular posterior wall fractures during surgery, adjust the angle of the acetabular cup to reduce forward leaning, the component is stable, and the patient was no-weight-bearing 4 weeks after surgery (Fig. 3). Because of severe comminution, another two patients got femoral head grafting to reconstruct the acetabulum, then the acetabulum was prepared with successive reamers to the minimum possible size fit to hold the acetabular shell (Fig. 4); A patient got anterior acetabular fracture during surgery. The original Betacup which applied was Unstable, after replacement with Pinnacle metal outer cup and fixation with two screws, the acetabular component was stable. The patients’ information, acetabular component, fracture characteristics are listed in the Table 1.

**Table 1:** The patients’ acetabular Component, Fracture Characteristics

Pt	1	2	3	4	5	6	7	8	9	10	11	12	13	14	15	16	17	18	19	20	21	22	23	24
Diagnosis	AS	AVN	AVN	AVN	AVN	AVN	AS	AS	DDH	DDH	OA	AVN	AVN	OA	AVN	AS	DDH	AS	AVN	DDH	DDH	DDH	AVN	AS
cup model	Combicup	Pinnacle	Betacup	Pinnacle	Pinnacle	T.O.P	Pinnacle	Pinnacle	comicup	Pinnacle	Betacup	Betacup	Pinnacle	Pinnacle	Betacup	Betacup	comicup	Combicup	Pinnacle	Pinnacle	Pinnacle	Pinnacle	Betacup	Pinnacle
Screws Used, n	5	4	1	3	9	1	3	1	1	1	0	0	1	14	0	1	3	3	2	5	3	2	0	2
steel plate	no	no	no	no	Yes	no	Yes	no	no	no	no	no	no	no	no	no	no	no	no	no	no	no	no	no
Fracture location	Anterior wall	Posterior wall	Medial wall	Posterior wall	Medial wall/ posterior wall/ posterio column	Medial wall	Posterior wall	Anterior column	Medial wall	Posterior wall	Posterior wall	Posterior wall	Medial wall	Posterior wall	Posterior wall	Anterior wall	Posterior wall	Anterior wall	Anterior wall	Posterior wall/ superior wall	Posterior wall	Posterior wall/ medial wall	Posterior wall	Anterior wall
Identified IAFs	Intraop	Intraop	Intraop	Intraop	Intraop	Postop	Intraop	Intraop	Intraop	Intraop	Intraop	Intraop	Intraop	Intraop	Intraop	Intraop	Intraop	Postop	Intraop	Intraop	Intraop	Intraop	Intraop	Intraop

According to the surgeon, 5 patients avoid weight-bearing for 4 weeks after surgery; the rest of the patients’ weight-bearing was advanced as tolerated after surgery.

## Results

Between 2015 to 2018, 24 patients sustained IAFs in 4888 primary THA, and all the acetabular fractures occurred in cases of uncemented acetabular components. The fracture rate associated with uncemented components was 0.49%.

In 17(70.8%) of the 24 cases, the fracture was noted during impaction of the real acetabular component; in 5(20.8%) cases, it was noted during initial hip dislocation and osteotomy Errors; in 1(4.1%) cases, it was noted during reaming. The location of the fracture was directly posterior wall in 11(45.8%) cases; anterior wall in 5(20.8%) cases, medial wall in 4(16.7%) cases, posterosuperior wall in 1(4.2%)case, posteromedial wall in 1(4.2%) case, posteromedial wall and posterior column in 1(4.2%) case, anterior column in 1(4.2%) case. Six patients(25%)with Ankylosing Spondylitis had fractures 4 in the anterior wall and 1 in the anterior column because of patients with hip joint fusion need a pre-osteotomy before the dislocation. Stability of the component was typically judged qualitatively according to the surgeon’s estimation of the quality of the “press-fit” and the absence of motion when pressing a probe on the periphery of the implanted cup.

Except two people were lost in the latest follow-up. The other patients’ latest HHS score increased from 30.8 ± 9.7 preoperatively to 90.2 ± 4.2 postoperatively. The difference value between pre-operation and post-operation is 59.7 ± 10.4(95% CI:55.1–64.3, *P*<0.05). They walked independently without any support. Overall, the score was excellent in 12 patients, good in 10 patients. (Table 2).

**Table 2:** Preoperative and postoperative HHS

	Minimum	Maximum	Mean	Standard deviation	
Preoperative HHS	13	48	30.8	9.7	t = 27.1
Preoperative HHS	82	97	90.2	4.2	*P*<0.05

Except the 2 patients were lost in the last follow-up, there was no loosening in any cup as evidenced by the absence of lucent lines in any zones in the X-rays. There was neither osteolysis nor any evidence of migration or change in the inclination of the component as evidenced by the serial X-rays done in the follow-ups. The fractures were united in all cases as evidenced by the healing of fracture lines previously seen in the initial X-rays. There was no subsidence or loosening of the femoral component as well in the serial radiographs.

**Fig. 5:**
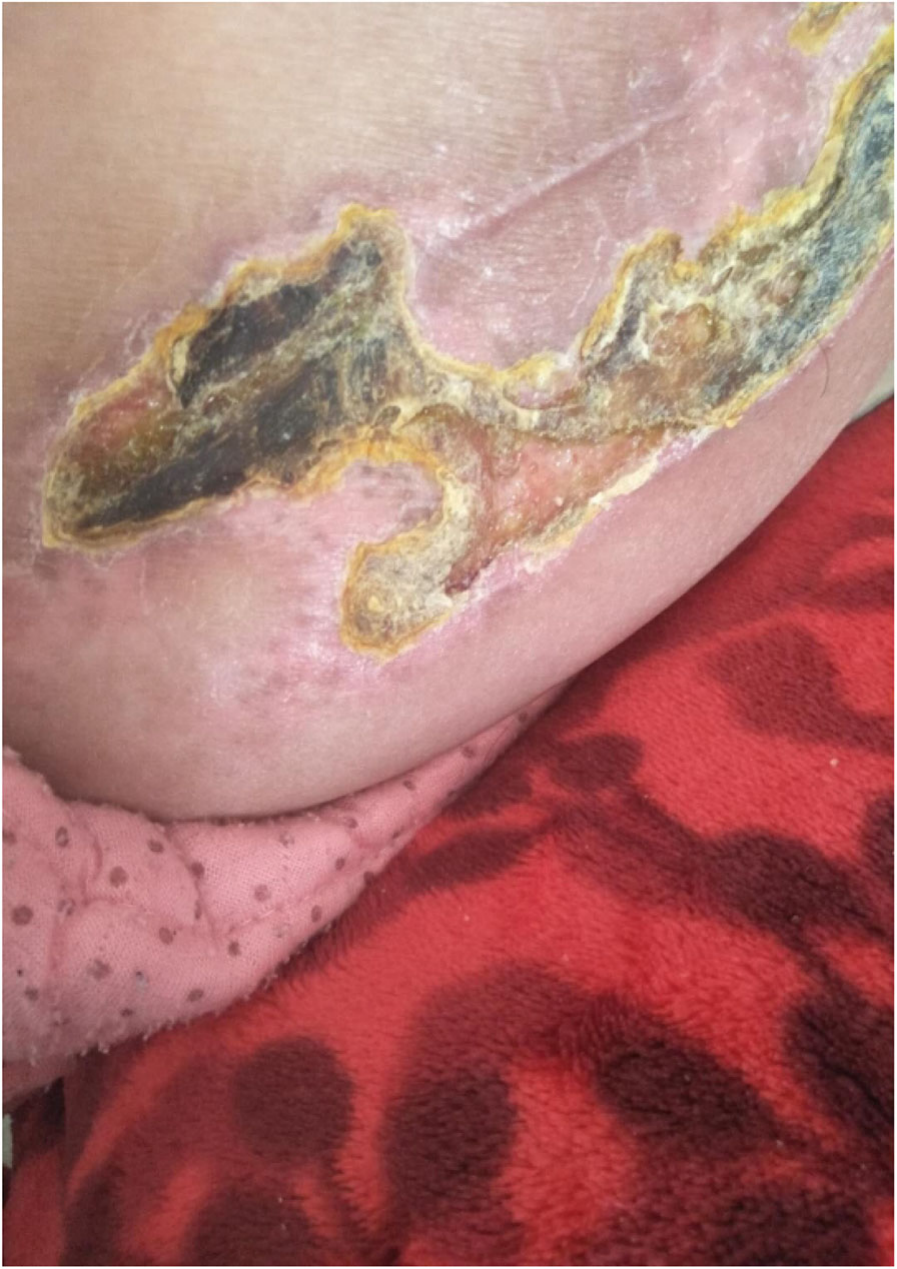
unhealed skin after left hip arthroplasty 45 days.

A patient had a large femoral trochanter split and was fixed with wire tension during the operation. The follow-up recovery was good and healed well. A patient had unhealed skin after left hip replacement 45 days. Follow-up skin healing was good after 1 year of invasive treatment (Fig. 5). A patient had a dislocation of the right hip joint 10 days after surgery. After failed manual reduction, the surgical incision and reduction were performed. The patient had good activity and no dislocation occurred 1 year after surgery. No other complications occurred.

## Discussion

IAF is a rare complication of the primary THA. Therefore, it has been difficult to accumulate a cohort large enough to study this problem. However, the incidence of IAFs has gradually increased in recent years [4, 7, 8]. Multiple clinical studies have suggested that uncemented THA’s are at a higher risk of acetabular fracture compared to their cemented counterparts [5, 12, 13]. With the wide use of uncemented acetabular components, as a joint surgeon will undoubtedly encounter IAFs in his surgical career. In this article, the fracture rate associated with uncemented components was 0.49%, which is close to 0.4% that the rate of fracture around the acetabular components when the uncemented acetabular components are implanted, as reported by Haidukewych and colleagues [6]. We introduce the treatment of our institution when encountering these problems which include methods and their prognosis.

Jonathon and colleagues [14] found that 2 patients with post-invasion posterior column were found by postoperative X-rays, and revision surgery was performed within 3 months; 1 patient was found intraoperatively with internal fixation, and the follow-up effect was good. In this series of our studies, the fracture of the posterior column occurred in patient5. During the operation, it was found that the internal fixation with steel plates and screws was performed. The follow-up effect was good after 4 years of operation, which verified this. Fractures occurring in the column should be found and treated in time.

The main difficulty in IAFs is to find it, estimate its level and obtain the stability of effective acetabular components to minimize the risk of aseptic loosening [15]. According to some authors, cable fixation can be used [15, 16] and internal fixation plates and screws [17] for adequate acetabular components stability. Tidemark and colleagues [18] found the use of strengthening plates in the presence of acetabular components fractures. However, in this cohort study, 23 patients found IAFs during operation, and were given timely screw internal fixation or plate and screws. Internal fixation and prognostic are good. The strengthening ring seems to provide better stability, but there is no significant difference in the prognosis or the incidence of complications between patients who use the screw alone and the combination of the strengthening ring and the screw. Haidukewych and colleagues [6] found that 21 patients with acetabular fractures were identified during the operation. All patients were identified for at least 2 years of follow-up or revision surgery. The results showed that all patients had good fracture healing except for 2 patients who were lost to follow-up. Complications occurred and bone growth was good. Our institution’s research verified this. For the IAFs found during the operation, the stability of the acetabular component was determined by screws or internal fixation plates, and timely treated during the operation. The prognosis is relatively good.

A patient had an autologous femoral head grafting to reconstruct the acetabulum, and the patient’s prognosis was good. In a cohort study of Sharkey and colleagues [4], 4 patients underwent autologous bone graft reconstruction to partially heal acetabular fractures.

Meek and colleagues [19] found that female patients are more prone to IAFs after primary total hip arthroplasty, which may be related to osteoporosis. In this study, the proportion of women with acetabular fractures during surgery was 67% which verified to that conclusion.

In this study, 5 patients had developmental dysplasia of the hip, femoral upward movement, acetabular hyperplasia, and a large number of osteophytes around the acetabulum, which caused difficulty in exposing the acetabulum and uneven acetabular grinding. THA in hip dysplasia is associated with increased risks of periprosthetic fractures owing to the disuse osteopenia and poor quality of the bone [20]. Therefore, when driving into the acetabular prosthesis, the external force required by the prosthesis holder is greater, and it is more likely to cause fractures. Therefore, it is necessary to fully expose the acetabulum during surgery to avoid blind and violent penetration into the prosthesis.

In this study, 6 patients with ankylosing spondylitis involving hip joints had severe hip flexion deformities. The ipsilateral pelvis tilted forward on the x-ray film, the acetabular contour, and the abduction angle changed, and the template measurement could not be accurate. Determine the placement position and angle of the prosthesis, causing the displacement of the prosthesis during the operation. At the same time, the IAFs is closely related to the surgeon’s operating skills, especially before the hip dislocation, because the hip joint fusion often requires pre-osteotomy. It is more difficult to handle. A patient in this study had a total fracture of the anterior wall of the acetabulum during the operation, and a full-thread screw was added to the fracture site. Fixation, acetabular stability during intraoperative testing, and good prognosis after follow-up.

Previously, it was thought that when acetabular sclerosis and ivory are susceptible to fractures under violence, osteoporosis is the main risk factor [6, 19, 21, 22]. In this study, two patients had severe bone Looseness, fractures of the posterior wall of the acetabulum during the operation, unstable test during the operation, and internal fixation with plate screws were given. The patient had a good prognosis and no joint dislocation occurred.

## Conclusion

IAFs are rare complications of primary THA, and most commonly occur during the implantation of the acetabular components. It is important to identify the fractures around the acetabular component during the operation. Prompt management is crucial for the patient’s prognosis. Patients with ankylosing spondylitis involving THA in hip patients. Must take care to prevent IAF during dislocation and pre-osteotomy.

## Data Availability

The datasets used and/or analyzed during the current study are available from the corresponding author on reasonable request.

## Abbreviations

IAF: Intraoperative acetabular fracture
THA: Total hip arthroplasty
AVN: Adult avascular necrosis
AS: Ankylosing spondylitis
DDH: Developmental dysplasia of the hip
OA: Osteoarthritis
